# Short and Long-Read Sequencing Survey of the Dynamic Transcriptomes of African Swine Fever Virus and the Host Cells

**DOI:** 10.3389/fgene.2020.00758

**Published:** 2020-07-28

**Authors:** Ferenc Olasz, Dóra Tombácz, Gábor Torma, Zsolt Csabai, Norbert Moldován, Ákos Dörmő, István Prazsák, István Mészáros, Tibor Magyar, Vivien Tamás, Zoltán Zádori, Zsolt Boldogkői

**Affiliations:** ^1^Institute for Veterinary Medical Research, Centre for Agricultural Research, Budapest, Hungary; ^2^Department of Medical Biology, Faculty of Medicine, University of Szeged, Szeged, Hungary

**Keywords:** African swine fever virus (ASFV), long-read sequencing, short-read sequencing, transcriptomics, direct RNA sequencing, full-length transcripts

## Introduction

Methodological breakthroughs in sequencing technologies have revolutionized transcriptome profiling in recent years. Currently, the next-generation short-read sequencing (SRS) and third-generation long-read sequencing (LRS) platforms are widely used in genome and transcriptome research. SRS can generate large numbers of sequencing reads with unprecedented speed; however, it cannot sufficiently cover high-complexity transcriptomes. LRS produces lower data coverage with a higher error rate, but it can overcome many of the drawbacks of SRS, including the inefficiency in distinguishing between transcription isoforms and identifying embedded and long transcripts. The combined use of these platforms and library preparation chemistries can generate high-quality and throughput data on full-length transcripts. LRS has been applied for the assembly of transcriptomic maps in several organisms (Sharon et al., 2013; Tilgner et al., 2015; Sessegolo et al., 2019; Yin et al., 2019; Zhao et al., 2019; Roach et al., 2020), including viruses (Tombácz et al., 2016, 2019; Balázs et al., 2017; Moldován et al., 2017; Prazsák et al., 2018; Depledge et al., 2019; O'Grady et al., 2019).

African swine fever is a highly lethal animal disease affecting pigs and wild boars. The causative agent of this disease is the large, double-stranded DNA virus, the African swine fever virus (ASFV), the only member of the *Asfarviridae* family (Mazur-Panasiuk et al., 2019). Because no effective vaccination is currently available against the virus, it is unarguably the largest economic threat to the global pig industry. The ASFV genome is 190 kbp long and contains more than 190 open reading frames (ORFs), although the exact numbers of genes and proteins are unknown (Alejo et al., 2018). Approximately 20 viral genes are believed to participate in transcription and mRNA processing (Rodríguez and Salas, 2013), whereas at least 17 genes play a role in the replication, repair, and modification of DNA (Yáñez et al., 1995; Dixon et al., 2013). Depending on the strain, ~30–50 genes are involved in the evasion of immune surveillance and in encoding virulence and host range factors (Chapman et al., 2008).

The temporal regulation of ASFV gene expression appears to be similar to that in poxviruses (Yáñez et al., 1995; Broyles, 2003; Chapman et al., 2008), in which four kinetic classes of genes have been described. The expression of immediate early and early genes precedes DNA replication, whereas the intermediate and late genes are generated subsequently to the onset of DNA replication (Rodríguez and Salas, 2013). The genome-wide ASFV transcriptome has recently been characterized by the Illumina SRS approach (Cackett et al., 2020). ASFV mRNAs have 5′ cap structures and 3′ poly(A) tails added by the viral capping enzyme complex and the poly(A) polymerase, respectively (Salas et al., 1986). ASFV replicates relatively well in porcine primary alveolar macrophages (PAMs) *in vitro*, although the sensitivity of naïve PAM culture to ASFV infection varies batch by batch (Olasz et al., 2019).

To provide a detailed transcription map about the transcription dynamics of the virus, we performed multiplatform sequencing using both SRS and LRS techniques. The presented dataset represent a key resource for studying the ASFV transcriptome at different time points after infection, and of the effect of infection on the host gene expression.

**Figure 1:**
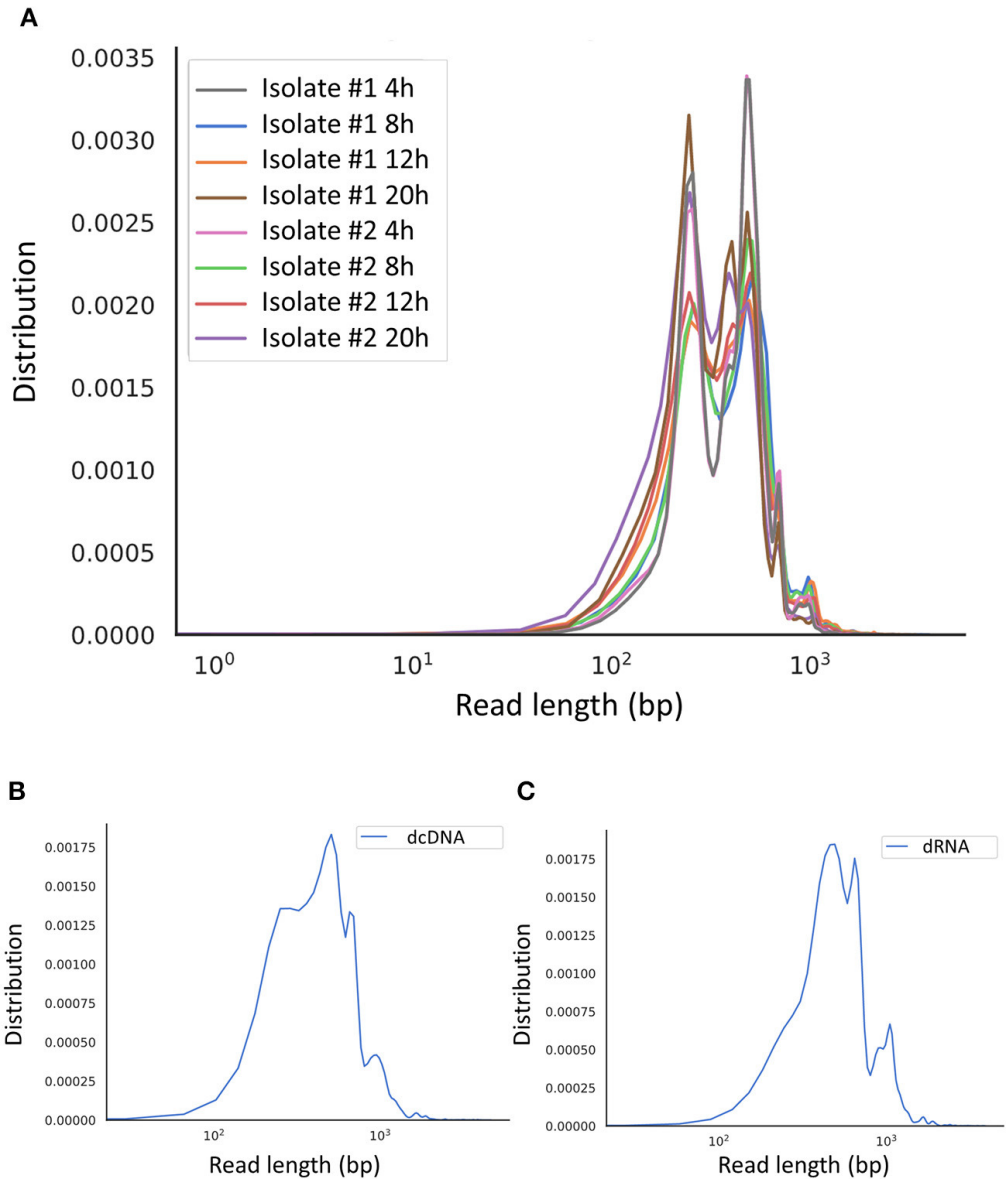
Aligned read length distribution. Line chart presentation of the average of aligned read lengths obtained via Nanopore sequencing. **(A)** Amplified cDNA sequencing at various time points. **(B)** Direct cDNA sequencing and **(C)** direct RNA sequencing using samples from multiple time points after the infection.

Regarding the SRS approach, the MiSeq instrument (Illumina) was used (Supplementary Figure 1 shows the coverage depth), whereas we applied the MinION portable sequencer from Oxford Nanopore Technologies (ONT) for full-length sequencing. The random-primed SRS library was run on a single MiSeq v3 flow cell, whereas three different ONT libraries [direct RNA sequencing (dRNA-Seq), direct cDNA sequencing (dcDNA-Seq) and amplified cDNA sequencing) were sequenced on three individual flow cells. Altogether the three LRS experiments resulted in 20,021,413 sequencing reads (Supplementary Table 1), of which 139,711 aligned to the viral genome (MN715134.1). The longest average read length was obtained using the dcDNA technique (1,299 bp). The average length for the amplified approach ranged between 598 and 1,017 bp, whereas the dRNA-Seq resulted in an average read length of 953 bp (Supplementary Table 1). More details about the length and quality of sequencing reads are presented in Supplementary Table 1 and Figure 1. The quality data from Illumina sequencing is presented in Supplementary Table 2.

## Methods

**Figure 2:**
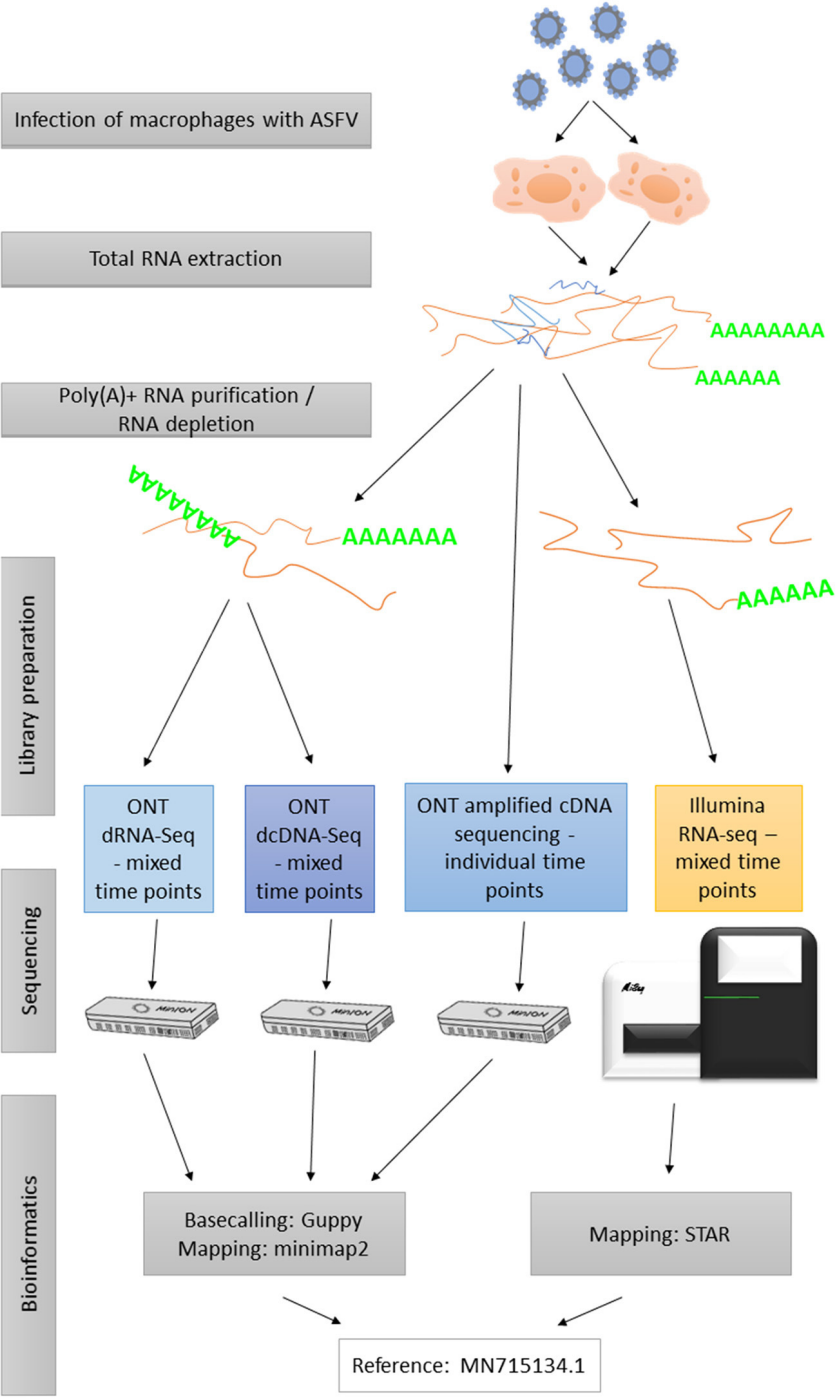
Flowchart diagram shows an overview of the methodological workflow of this study.

The experimental design utilized in this study is shown in Figure 2. The applied reagents are listed in Supplementary Table 3.

### Cells and Viruses

Fresh swine pulmonary macrophage (PAM) cells were harvested following the OIE Manual's instructions (Office International des Epizooties (OIE), 2019). The cells were grown in RPMI 1640 containing L-glutamine (Lonza) medium supplemented with 10% fetal bovine serum (Euro Clone), 1% Na-pyruvate (Lonza), 1% antibiotic-antimycotic solution (Thermo Fisher Scientific), and 1% non-essential amino acid solution (Lonza) at 37°C in a humidified atmosphere containing 5% CO_2_. The highly virulent Hungarian ASFV isolate ASFV_HU_2018 (ID Number: MN715134) was used for infection. The infectious titer of the serially diluted viral stock was calculated in PAMs using an immunofluorescence (IF) assay as described previously (Olasz et al., 2019). All work with the infectious virus was conducted at the biosafety level 3 (BSL3) laboratory of the Institute for Veterinary Medical Research, Center for Agricultural Research following all current EU regulations (European Commission, 2020).

### Infection

PAMs were cultivated in 6-well plates at a density of 3.3 × 105 cells and infected at a multiplicity of infection of 10 at 4 h after cell seeding. Supernatant was replaced with fresh medium at 1 h post-infection (p.i.), and infected cells were harvested at 4, 8, 12, and 20 h p.i. Mock-infected control cells were also harvested.

### RNA Purification

#### Isolation of Total RNA

A NucleoSpin® RNA (Macherey-Nagel) kit was used for RNA purification following the manufacturer's instructions. In brief, the supernatant was removed from all wells of the 6-well plate, and 2 × 106 cells were lysed with RA1 lysis buffer and β-mercapthoethanol solution. Then, the lysates were transferred to NucleoSpin filters. After centrifugation (11,000 × g, 1 min), 70% ethanol were added to the lysates. The solutions were transferred to the columns, and after centrifugation, the membranes were washed with MDB buffer. After repeated washing, DNase reaction mixture were added, and the membranes were incubated at room temperature for 15 min. The membranes were washed with RAW2 and RA3 buffer, and the tubes were centrifuged at 11,000 × g for 30 s. Finally, RA3 buffer were added, and the tubes were centrifuged at 11,000 × g for 2 min. RNA was eluted with RNase-free H_2_O and centrifuged at 11,000 × g for 1 min. All buffers were supplied from the kit.

#### Purification of Polyadenylated RNA

For the various Nanopore sequencing approaches, the polyA(+) RNA fraction of total RNA was isolated using the “Spin Columns method” from the Oligotex mRNA Mini Kit (Qiagen).

#### Ribosomal RNA Depletion

For Illumina sequencing, rRNA was eliminated from the total RNA samples using the RiboMinus™ Eukaryote System v2 (Invitrogen).

### Library Preparation for Nanopore Sequencing

#### Direct RNA Sequencing—Using Samples From Mixed Time Points

The dRNA-Seq method using a direct RNA sequencing kit (SQK-RNA002, Version: DRS_9080_v2_revK_14Aug2019) from ONT was used for amplification-free sequencing. This approach is highly recommended to explore special features of native RNA (e.g., modified bases) and avoid potential biases associated with reverse transcription (RT) or PCR. Total RNA from eight samples (two parallel experiments from 4, 8, 12, and 20 h p.i.) was mixed together, and then the polyA(+) fraction of RNA was purified from the sample mix. One hundred nanograms from the polyA-tailed RNA were mixed with RT Adapter (oligo dT-containing T10 adapter), RNA CS (both from the Nanopore kit; the latter was used to monitor the sequencing quality), NEBNext Quick Ligation Reaction Buffer, and T4 DNA ligase [both from New England Biolabs (NEB)]. The mixture was incubated for 10 min at room temperature, and then RT was conducted to generate first-strand cDNA using dNTPs (NEB), 5 × first-strand buffer, DTT (both from Invitrogen SuperScript III) and UltraPure™ DNase/RNase-Free distilled water (Invitrogen), and then the sample was mixed with SuperScript III enzyme (Thermo Fisher Scientific). RT was performed in a Veriti cycler (Applied Biosystems) at 50°C for 50 min, and the reaction was subsequently terminated at 70°C for 10 min. RNA-cDNA hybrids were purified using Agencourt RNAClean XP Beads [1.8 bead ratio (BR)]; Beckman Coulter], washed with freshly prepared 70% ethanol, and eluted in UltraPure™ nuclease-free water. The sample was then ligated to the RNA adapter (RMX from the ONT kit) at room temperature for 10 min using NEBNext Quick Ligation Reaction Buffer, T4 DNA ligase, and nuclease-free water. The ligation reaction was followed by a final purification step using XP Beads (1.0 BR). Samples were washed with wash buffer (ONT) and eluted in elution buffer (ONT). After Qubit measurement, 100 fmol from the library were loaded onto a Flow Cell.

#### Direct cDNA-Seq—From Mixed Time Points

Viral and host transcripts were also sequenced on a MinION sequencer following the instructions of the direct cDNA sequencing kit (SQK-DCS109; Version: DCS_9090_v109_revJ_14Aug2019; ONT). This protocol is based on strand switching, and it is highly optimal for the generation of full-length cDNA for the identification of potential novel transcript isoforms without potential PCR bias. The starting material was 100 ng of a poly(A)+ RNA mixture from various time points of infection (4, 8, 12, and 20 h p.i.). An oligo dT-containing VN primer (VNP) and dNTPs (10 μM) were added to the RNA. After 5 min of incubation at 65°C, the following components were added: 5 × RT buffer (from the Maxima H Minus Reverse Transcriptase kit, Thermo Fisher Scientific), RNaseOUT™ (Thermo Fisher Scientific), strand switching primer from the ONT kit and nuclease-free water. This mixture was pre-heated at 42°C for 2 min, and Maxima H Minus Reverse Transcriptase was added. RT was conducted at 42°C for 90 min, and finally, the reaction was stopped by incubation at 85°C for 5 min. RNase Cocktail Enzyme Mix (Thermo Fisher Scientific) was used to degrade the RNA in the sample. Incubation was performed at 37°C for 10 min. Before the second-strand synthesis, the sample was cleaned using AMPure XP Beads (0.8 BR) (Beckman Coulter). Then, LongAmp Taq Master Mix (NEB) was used to synthesize second-strand cDNA using the PR2 primer (ONT). Samples were incubated using the following “only one cycle protocol:” denaturation at 94°C for min, annealing at 50°C for 1 min, and elongation at 65°C for 15 min. The double-stranded cDNAs were purified using AMPure XP method (0.8 BR). NEBNext Ultra II End-prep reaction buffer and NEBNext Ultra II End-prep enzyme mix (both from NEB) were added to each sample. This end repair process was performed at 20°C for 5 min, followed by a 5-min incubation at 65°C. Enzymes and buffers were removed from the reaction using the AMPure XP purification method (1.0 BR), and then the sample was subjected to adapter ligation. The ONT Adapter Mix and Blunt/TA Ligation Master Mix (NEB) were mixed with each sample, and the mixture was incubated at room temperature for 10 min. A final AMPure XP purification (0.4 BR) was conducted to remove any excess proteins, nucleotides, and salts from the DNA library. Adapter Bead Binding Buffer was used to wash the beads, and the library was eluted using elution buffer (both from the Nanopore kit). The samples were quantified using Qubit, and then 200 fmol from the sample were loaded on two MinION Flow Cells.

#### Amplified cDNA-Sequencing—From Different Time Points

Samples from each time point (4, 8, 12, 20 h p.i. and the mock infected cells) were sequenced by the ONT MinION device using the cDNA-PCR Barcoding protocol (SQK-PCS109 and SQK-PBK004; Version: PCSB_9086_v109_revK_14Aug2019). This protocol is recommended to identify and quantify full-length transcripts, discover novel isoforms, and splice variants and fusion transcripts from a low amount of starting material (total RNA) to generate large amounts of cDNA data. Approximately 50 ng of each of the samples were used for library preparation. VNP and dNTPs were added to the RNA and incubated at 65°C for 5 min. The strand-switching buffer mixture (RT buffer, RNaseOUT, nuclease-free water, and SSP) was added to the samples, which were incubated at 40°C for 2 min. RT was conducted by adding Maxima H Minus Reverse Transcriptase at 42°C for 90 min. The enzyme was inactivated by increasing the temperature to 85°C for 5 min. LongAmp Taq Master Mix, one of the Low Input barcode primers (LWB01-12, from the ONT's SQK-PBK004 kit, Supplementary Table 4), and nuclease-free water were included in the RT reaction mixture. Supplementary Table 5 shows the PCR conditions. PCR products were treated with exonuclease (NEB), and the mixture was then incubated at 37°C for 15 min, followed by 80°C for 15 min. AMPure Beads (0.8 BR) was used for purification, and the clean sample was eluted.

### Library Preparation for Illumina Sequencing

A NEXTflex® Rapid Directional qRNA-Seq Kit (PerkinElmer) was used to sequence the whole ASFV transcriptome via a conventional short-read approach. We used 25 ng of an rRNA-depleted RNA mixture (4, 8, 12, and 24 h p.i.) as the starting material. The first step was enzymatic fragmentation of the RNA using NEXTflex® RNA Fragmentation Buffer. The reaction was conducted at 95°C for 10 min followed by first-strand cDNA synthesis. First, NEXTflex® First Strand Synthesis Primer was added to the reaction mixture, which was heated at 65°C for 5 min and then subsequently placed on ice. NEXTflex® Directional First Strand Synthesis Buffer and Rapid Reverse Transcriptase were then added. RT was performed using the following program: incubation at 25°C for 10 min, heating at 50°C for 50 min, and termination at 72°C for 15 min. This step was followed directly by second-strand cDNA synthesis via the addition of NEXTflex® Directional Second Strand Synthesis Mix (with dUTPs) at 16°C for 60 min. The product was cleaned using AMPure Beads (1.8 BR). Resuspension buffer (NEXTflex® Kit) was used for the final elution. Polyadenylation of the double-stranded cDNAs was performed using NEXTflex® Adenylation Mix at 37°C for 30 min. The reaction was terminated by heating at 70°C for 5 min. Molecular Index Adapters (from the Kit) were ligated to the sample at 30°C (10 min) using the NEXTflex® Ligation Mix. Prior to amplification, each sample was washed with AMPure Beads (0.8 BR). First, NEXTflex® Uracil DNA Glycosylase was mixed with the sample, which was incubated at 37°C for 30 min, followed by heating at 98°C for 2 min. The sample was placed on ice, and the following components were added: PCR Master Mix, qRNA-Seq Universal forward primer, and qRNA-Seq Barcoded Primer (sequence: AACGCCAT; all from the kit). The samples were amplified according to the protocol summarized in Supplementary Table 6. The PCR products were washed with AMPure Beads (0.8 BR), and followed by a second purification.

### Sequencing on the Illumina MiSeq Instrument

The sequencing-ready library (12 pM) was loaded onto a flow cell from Illumina MiSeq Reagent Kit v3 (150-cycle format, 2 × 75 bp) and sequenced on a MiSeq sequencer.

### Read Processing

#### ONT Sequencing

Guppy software v3.4.5 (ONT) was used for base calling from MinION data. The raw reads were aligned to the ASFV reference genome (NCBI Nucleotide accession: MN715134.1) using the minimap2 software suite (Li, 2018) with the following options: -ax splice -Y -C5 –cs. SeqTools scripts were used to obtain the quality information.

#### Illumina Sequencing

Raw reads were trimmed using Cutadapt software (Martin, 2011), the aforementioned viral reference genome was indexed using STAR aligner v2.7.3a (Dobin et al., 2013) with the following settings: –genomeSAindexNbases 8, followed by the mapping of the reads with default options. Samtools (Li et al., 2009) was used to sort the sam files and to generate and index the BAM files. The Qualimap v2.2.1 application (García-Alcalde et al., 2012) was used to generate quality information from the Illumina dataset.

### Code Availability

**Guppy v3.4.5:** http://community.nanoporetech.com/downloads?fbclid=IwAR2IchRL4gDnfA6h996UkN4vS5pbBu6rUtKVFX3a TiBHsWFknglQ6FyvPkg

**minimap2:** https://github.com/lh3/minimap2

**STAR:** https://github.com/alexdobin/STAR

**samtools:** https://github.com/samtools/samtools

**SeqTools**: https://github.com/moldovannorbert/seqtools.

## Technical Validation

The total RNA, polyA(+) RNA, and rRNA-depleted samples; generated cDNAs; and final sequencing libraries were quantified by a Qubit 4 Fluorometer using Qubit RNA Broad-Range, High Sensitivity RNA, and High Sensitivity dsDNA Assay Kits. The Agilent TapeStation 4150 system was applied to detect the integrity of total RNA and perform a quality check of the Illumina libraries. In the present study, RNAs samples with RIN ≥ 9.4 were subjected to construct the sequencing libraries (ode Avail Supplementary Figure 2).

## Data Re-Use

To our best knowledge, no data on the ASFV transcriptome are available; therefore, this dataset was primarily generated to characterize the RNA profile of the virus. The dataset can be used to detect RNA isoforms, including length (alternative 3′ and 5′) variants, monocistronic, bicistronic, polycistronic, and complex transcripts, and to discover transcriptional overlaps and the complexity of the genetic regulation of ASFV. Nanopore dataset allows a time-course evaluation of the full-length transcriptomes of both the virus and host. The published BAM files contain the reads mapped to the MN715134.1 reference genome. BAMs [using samtools and bedtools (Quinlan and Hall, 2010)] can be converted to FastQ files, which extend the potential usage of data; e.g., they can be aligned to host genome. BAM files can be analyzed using various bioinformatics tools, such as samtools, bedtools, or the Genome Analysis Toolkit (Van der Auwera et al., 2013). The Nanopore data generated with different library preparation approaches can be compared to analyze the differences between the sequencing chemistries, as well as the effect of RT and PCR reactions on the length and quality of the reads. The provided dataset is also useful for comparing the performance of the utilized sequencing platforms. Tombo tool (Stoiber et al., 2017) can be used to identify CpG methylation patterns and base modifications (e.g., A to I editing) from raw (fast5) Nanopore sequencing data, or the EpiNano (Liu et al., 2019) algorithm can be applied to detect m6A RNA modifications. The dataset can be further analyzed using various bioinformatics program packages or visualized using softwares [e.g., Integrative Genomics Viewer (Robinson et al., 2011), Savant Genome Browser (Fiume et al., 2010), Geneious (Kearse et al., 2012)].

## Data Availability Statement

The datasets generated for this study can be found in the European Nucleotide Archive - accession number PRJEB36723.

## Ethics Statement

Animals were euthanized in the animal facilities of ATK ÁOTI where all methods were performed in accordance with the relevant guidelines and regulations following the protocol approved by the Government Agency of Pest County (PE/EA/1474-7/2017). All methods were performed in accordance with the relevant guidelines and regulations following a protocol approved by the ATK ÁOTI Institutional Animal Care and Use Committee.

## Author Contributions

FO participated in the infection experiments, the purification of the total RNA from the virus, and with writing the manuscript. DT performed the MinION sequencing (dynamic dataset) and the Illumina sequencing, participated in data analysis, and wrote the manuscript. GT generated the ONT dRNA sequencing libraries and conducted data handling and processing. ZC conducted MinION direct cDNA sequencing and the Illumina sequencing. NM carried out bioinformatics analysis. ÁD performed the isolation of poly(A)+ RNAs and the rRNA removal from total RNAs. IP participated in the purification of poly(A)+ RNA and data handling. IM conducted the infection experiments, purified the viral total RNA samples. TM prepared the pulmonary macrophages. VT participated in the infection experiments and the purification of the total RNA from the virus. ZZ designed the research plan and wrote the manuscript. ZB conceived and designed the experiments, managed the study, and wrote the manuscript. All authors read and approved the final paper.

## Conflict of Interest

The authors declare that the research was conducted in the absence of any commercial or financial relationships that could be construed as a potential conflict of interest.
