# Supplementary material for: Short and Long-Read Sequencing Survey of the Dynamic Transcriptomes of African Swine Fever Virus and the Host Cells
Source: Front Genet. 2020 Jul 28;11:758. doi: 10.3389/fgene.2020.00758 (PMC7399366; doi:10.3389/fgene.2020.00758)
Supplement: Supplementary file 7 [file Data_Sheet_1.docx]

Supplementary Material

# Supplementary Figures and Tables

## Supplementary Figures


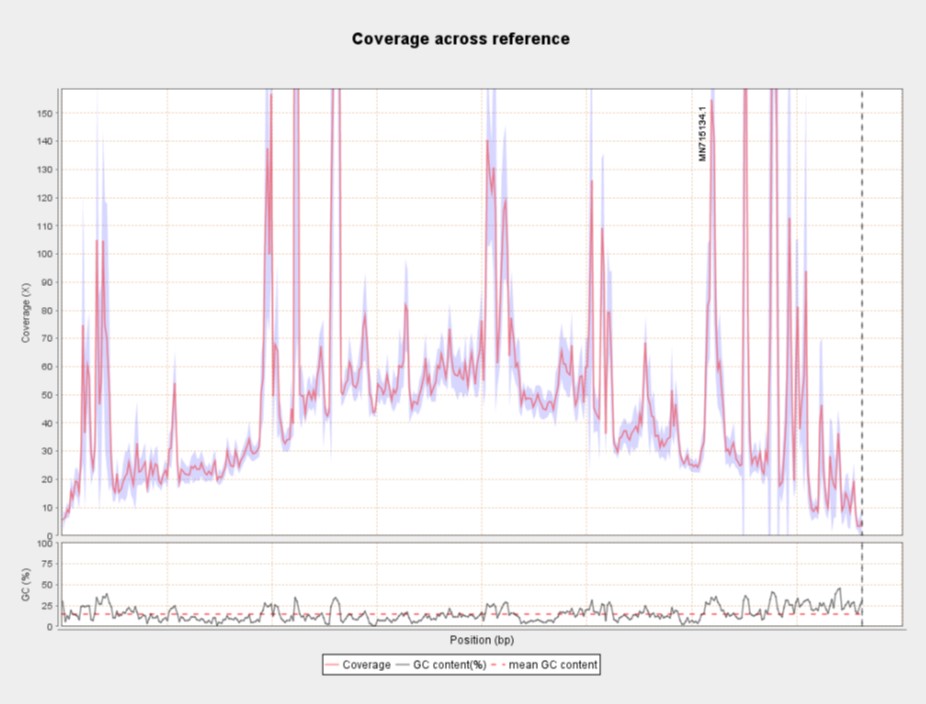


**Supplementary Figure 1.** **Illumina read depth along the African swine fever virus genome** (MN715134.1). The G+C content of the virus is also shown at the bottom of the figure.

**
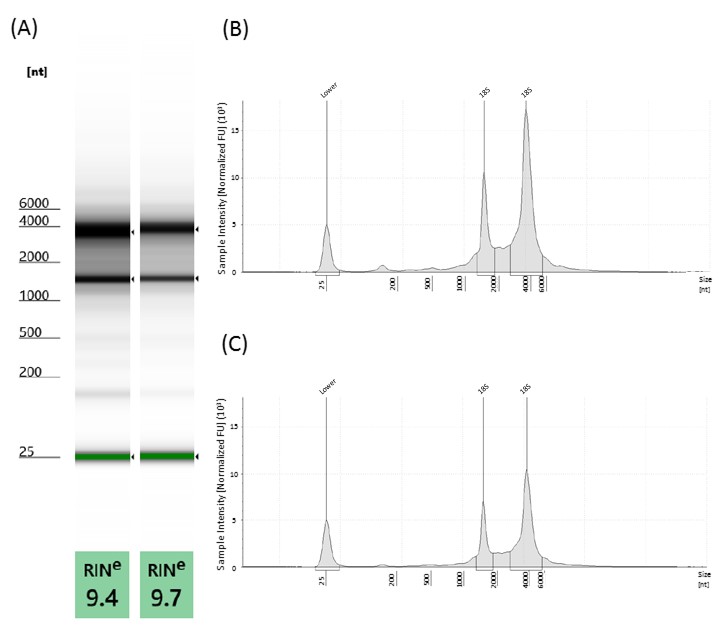
**

**Supplementary Figure 2.** **Total RNA analysis was conducted using the TapeStation 4150 system.** High quality [RNA integrity number (RIN) = 9.4–9.7]. RNA samples were used for sequencing. (a) ScreenTape gel image shows the separation profile of two total RNA samples (Animal #1 8 h and Animal #2 12 h) along with the RINs. Panels (b) and (c) show representative electropherograms of total RNA from Animal #1 8 h and Animal #2 12 h, respectively. The electropherograms show ribosomal 28S and 18S RNA peaks and the lower marker.

## Supplementary Tables

**Supplementary Table 1. Detailed statistics of the Nanopore sequencing reads mapped to the African swine fever virus genome for each run.** In this study, we generated full-length transcripts of ASFV. The utilized nanopore-based cDNA sequencing approaches yielded 20,021,413 reads, 14,538,950 of which were generated by the amplified library preparation protocol. The ratio of viral transcripts (mapped reads) fell between 0.23-0.68%, which was dependent on the stage of the viral life cycle at the examination period, as well as on the library preparation method. The sequencing method affected read length in the following way: the non-amplified direct RNA and cDNA sequencing methods produced longer reads (dRNA mapped reads are the longest) compared to the amplified technique due to the fact that PCR produces more copies from the shorter transcripts. In our experiments, the dRNA-seq approach produced a relatively high error rate, which has also been described by others (Wongsurawat et al. 2018).

Wongsurawat, T., Jenjaroenpun, P., Wassenaar, T. M., Wadley, T. D., Wanchai, V., Akel, N. S., et al. (2018). Decoding the Epitranscriptional Landscape from Native RNA Sequences. *bioRxiv*. 487819; doi: 10.1101/487819

**Supplementary Table 2. Summary statistics of the Illumina reads aligning to ASFV genome.** The statistics were generated using Qualimap v2.2.1. Illumina sequencing yielded 69,068 virus-specific reads with a mean read length of 67,52 bps. The mean insert size was 445.27 bps. The median (p50) and interquartile boundary values (p25, p75) for insert lengths, the quality of the mapped reads, as well as the sequencing errors (insertions, deletions) were also calculated. The number of duplicated reads was 36.12%, which indicates that certain genes are over- or underrepresented (genes with extremely low and high expression values, respectively).

**Supplementary Table 3. List of reagents and chemistries used in this work.** Abbreviations: prep, preparation; DW, distilled water; Tech, technologies; ONT, Oxford Nanopore Technologies; Sci, Scientific; acDNA, amplified cDNA

**Supplementary Table 4. Barcode identifiers and sequences used for library preparation for the amplified cDNA sequencing approach.** Amplified cDNA libraries were run on a single flow cell, therefore we labelled them with barcodes during library preparation process.

**Supplementary Table 5. The protocol used for the amplification of the cDNA samples.** PCR was carried out according to the ONT MinION Amplified cDNA-sequencing protocol, with the following modifications: 14 PCR cycles were applied for the amplification of cDNA products. This cycle number was optimal (within the suggested 11-18 cycle numbers) for the protocol recommended test PCR reaction (enough cDNA amount for the downstream applications without high amount of primer dimers and other aspecific products). A relatively long extension step (4 min) was set for the reactions which is required by the multiplication/amplification of long transcripts. The reactions were carried out in a Verity thermal cycler (Applied Biosystems).

**Supplementary Table 6. Table shows the PCR parameters used for Illumina library preparation.** Amplification of cDNA for the Illumina MiSeq platform was carried out using the PerkinElmer’s NEXTflex® Rapid Directional qRNA-Seq™ Kit. For Illumina short-read sequencing, a shorter extension [60 sec; (compare to the MinION)] and 15 cycles were applied. The Applied Biosystems’ Verity cycler was used for the PCR amplification.
